# Electromagnetic Multipole Theory for Two-Dimensional Photonics

**DOI:** 10.1021/acsphotonics.4c02194

**Published:** 2025-02-19

**Authors:** Iridanos Loulas, Evangelos Almpanis, Minas Kouroublakis, Kosmas L. Tsakmakidis, Carsten Rockstuhl, Grigorios P. Zouros

**Affiliations:** † Section of Condensed Matter Physics, National and Kapodistrian University of Athens, Panepistimioupolis, 157 84 Athens, Greece; ‡ Institute of Nanoscience and Nanotechnology, NCSR “Demokritos”, Patriarchou Gregoriou and Neapoleos Street, Ag. Paraskevi, 153 10 Athens, Greece; ¶ School of Informatics, Aristotle University of Thessaloniki, 541 24 Thessaloniki, Greece; § Institute of Theoretical Solid State Physics, Karlsruhe Institute of Technology, 76131 Karlsruhe, Germany; ∥ Institute of Nanotechnology, Karlsruhe Institute of Technology, 76131 Karlsruhe, Germany; ⊥ School of Electrical and Computer Engineering, National Technical University of Athens, 157 73 Athens, Greece

**Keywords:** Active media, anisotropic, electromagnetic scattering, multipole decomposition, two-dimensional

## Abstract

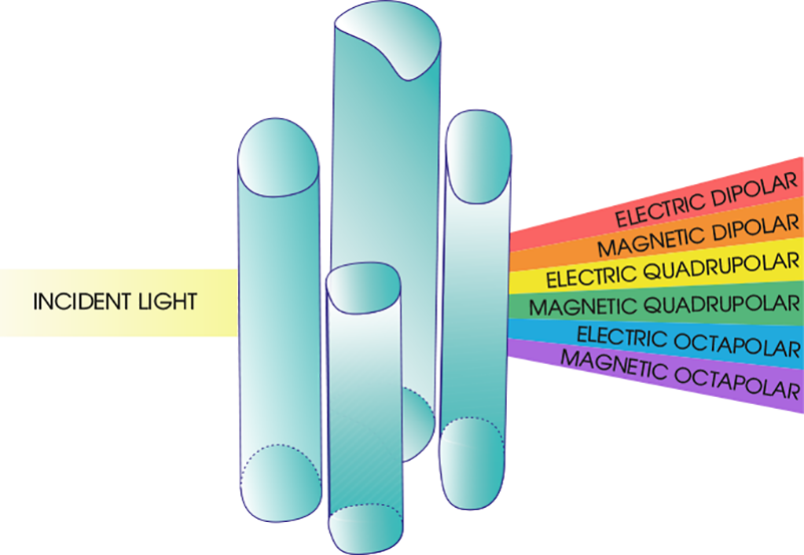

We develop a full-wave electromagnetic (EM) theory for calculating the multipole decomposition in two-dimensional (2-D) structures consisting of isolated, arbitrarily shaped, inhomogeneous, anisotropic cylinders or a collection of such. To derive the multipole decomposition, we first solve the scattering problem by expanding the scattered electric field in divergenceless cylindrical vector wave functions (CVWFs) with unknown expansion coefficients that characterize the multipole response. These expansion coefficients are then expressed via contour integrals of the vectorial components of the scattered electric field evaluated via an electric field volume integral equation (EFVIE). The kernels of the EFVIE are the products of the tensorial 2-D Green’s function (GF) expansion and the equivalent 2-D volumetric electric and magnetic current densities. We validate the theory using the commercial finite element solver COMSOL Multiphysics. In the validation, we compute the multipole decomposition of the fields scattered from various 2-D structures and compare the results with alternative formulations. Finally, we demonstrate the applicability of the theory to study an emerging photonics application on oligomer-based highly directional switching using active media. This analysis addresses a critical gap in the current literature, where multipole theories exist primarily for three-dimensional (3-D) particles of isotropic materials. Our work enhances the understanding and utilization of the optical properties of 2-D, inhomogeneous, and anisotropic cylindrical structures, contributing to advancements in photonic and meta-optics technologies.

## Introduction

The study of optical particles and their interactions with light is fundamental in advancing various technological areas such as photonics and meta-optics. Multipole decomposition provides a robust framework for understanding and controlling these interactions. It involves decomposing the electromagnetic (EM) field scattered by an optical particle or by a system of such particles into a multipolar series. The individual terms in the series are distinct and correspond to dipolar, quadrupolar, octupolar, and higher-order terms. This decomposition offers profound insights into the scattering behavior and reveals the underlying physics at subwavelength scales.

Multipole analysis has been widely employed to investigate a broad spectrum of cutting-edge phenomena in three-dimensional (3-D) structures, including meta-atoms for the manipulation of EM waves,^1^ design of metadimers with unique optical properties,^2^ Kerker scattering to control the directionality of light via multipolar interferences,^3^ zero optical backscattering from single nanoparticles,^4^ directed light emission through multipolar interferences,^5^ coupling enhancement of multipole resonances via optical beams,^6^ highly transmissive metasurfaces for polarization control,^7^ generalized Kerker effects in meta-optics,^8^ selective excitation of multipolar resonances via cylindrical vector beams,^9^ magnetic switching of Kerker scattering,^10^ dynamic control for invisibility to superscattering switching,^11^ excitation of higher-order multipolar modes via displacement resonance,^12^ and more complex 3-D structures consisting of meta-atoms or meta-molecules such as metasurfaces and metagratings^13−17^ as well as dense ensembles of plasmonic nanoparticles.^18^

On the other hand, two-dimensional (2-D) structures, such as nanowires, long rods, oligomers, or metalattices/metagratings thereof,^19^ exhibit unique optical properties that can be harnessed for advanced technological applications. Multipole analysis of cylindrical structures has a plethora of applications, including efficient magnetic mirrors,^20^ scattering invisibility in all-dielectric nanoparticles,^21^ active tuning of directional scattering of magneto-optical structures,^22^ polarization manipulation in cylindrical metalattices,^23^ optical beam steering control via dielectric diffractionless metasurfaces and diffractive metagratings,^24^ tuning of toroidal dipole resonances in all-dielectric nanocylindrical metamolecules,^25^ reconfigurable metalattices via magneto-optically coated rods,^26^ arbitrary beam steering exploiting transverse Kerker scattering,^27^ and extreme nonreciprocal scattering in asymmetric gyrotropic cylindrical trimers.^28^

Complete theories for multipole decomposition have been developed exclusively for 3-D particles of isotropic materials. These theories include expansion of the EM field in spherical eigenvectors,^29^ exact expressions for source dipolar moments as well as for dipoles that radiate a definite polarization handedness,^30^ the derivation of exact multipole moments beyond the subwavelength limit,^31^ an efficient multipole decomposition procedure using Lebedev and Gaussian quadrature,^32^ and exact multipole moments for magnetic particles of arbitrary shape and size.^33^

In this work, we provide, for the first time, a full-wave theoretical framework for the derivation of multipole decomposition in arbitrary 2-D inhomogeneous gyrotropic structures composed of either an isolated scatterer or collections of such. This framework is undoubtedly significant for various fields of photonics, including nanoparticle engineering,^34^ dielectric metasurfaces,^35^ superscattering enhancement,^11^ and Mie-tronics,^36^ to name a few. In addition, being an extensive theory, it complements existing theories on multiple scattering by nanoparticle structures^37,38^ applied for photonics applications. First, the scattered electric field **E**^sc^(**ρ**)—where **ρ** = (ρ, φ) is the position vector in polar coordinates—is expanded in divergenceless cylindrical vector wave functions (CVWFs) with unknown expansion coefficients *A*_*m*_, *B*_*m*_ that determine the multipole response. *A*_*m*_ and *B*_*m*_ are next expressed via contour integrals with integrand functions considering the vectorial components of **E**^sc^(**ρ**). In sequence, **E**^sc^(**ρ**) is calculated through a 2-D electric field volume integral equation (EFVIE) whose kernels are the products of the tensorial 2-D Green’s function (GF) expansion  and the equivalent 2-D volumetric electric current density **J**_eq_(**ρ**) and magnetic current density **M**_eq_(**ρ**). This scheme allows for the determination of *A*_*m*_ and *B*_*m*_ via 2-D volumetric integrations in the gyrotropic region of the scatterers.

We exhaustively validate our theory, first by calculating the multipole decompositions of various 2-D structures whose scattered fields have been obtained from the full-wave Maxwell solver COMSOL. We also compare our results to those obtained with the exact formulation for isotropic and gyrotropic circular cylinders,^39^ with the Mathieu functions method for elliptical cylinders,^40^ with the coupled-field volume integral equation–cylindrical Dini series expansion (CFVIE-CDSE) method for core–shell circular cylinders,^41^ and with the method of auxiliary sources (MAS) for circular core–elliptical shell and circular dimer cylinders.^42^ In all of these comparisons, excellent agreement is observed. The main advantage that our approach provides, compared to the above-mentioned ones^39−42^ used for method verification, is that it allows the theory to be implemented in any general-purpose solver, such as COMSOL, so as to perform the multipole decomposition on complicated structures composed of scatterers of noncanonical shapes with anisotropic material properties. This is a burdensome task for semianalytical techniques, since their development requires cumbersome manipulations, while their applicability is limited, mainly, to canonical shapes or perturbed variants.

The paper is organized as follows: In the first section, we develop the theoretical framework, and then we provide various validation examples, after which there is a discussion on a photonics application on oligomer-based highly directional switching using active media. The final section concludes the paper.

## Theory

### Multipole Decomposition for an Isolated Scatterer

**Figure 1:**
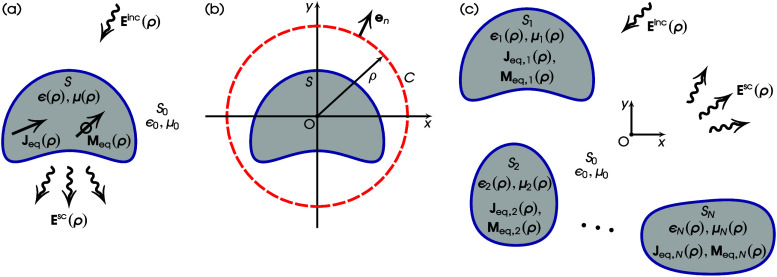
(a) Problem to be solved. (b) Integration path that encloses the scatterer. (c) Multiple scattering by an arrangement of scatterers.

The configuration problem is depicted in Figure 1a. An incident electric field **E**^inc^(**ρ**) impinges on a 2-D isolated scatterer *S* described by an inhomogeneous, gyrotropic material with constitutive parameters **ϵ**(**ρ**) and **μ**(**ρ**), given by
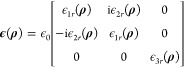

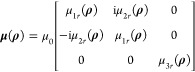
1where ϵ_0_ and μ_0_ are the free space permittivity and permeability, respectively. The background medium *S*_0_ is free space. Applying the volume equivalence theorem,^43^ 2-D volumetric electric  and magnetic  current densities—where **ρ** ∈ *S*,  is the unity dyadic, and **E**(**ρ**) and **H**(**ρ**) are the total electric and magnetic fields—are induced inside the scatterer that in turn produce the scattered electric field **E**^sc^(**ρ**) in *S*_0_. The adopted time dependence is exp(iω*t*). Given the solution of the total EM field **E**(**ρ**), **H**(**ρ**) via any desirable method, the purpose is to decompose **E**^sc^(**ρ**) into a series of electric and magnetic multipoles, i.e., to provide a theory for the multipole decomposition of any 2-D scattering problem.

For a 2-D problem, the multipoles are conveniently calculated when **E**^sc^(**ρ**) is expanded in CVWFs by
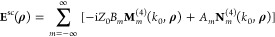
2where *A*_*m*_ and *B*_*m*_ are the unknown expansion coefficients to be evaluated,  is the free-space impedance, *k*_0_ = ω(ϵ_0_μ_0_)^1/2^ is the free-space wavenumber, and  and  are the CVWFs of the fourth kind, which for infinitely long configurations—i.e., ∂/*∂z* = 0—are given by^41,44^
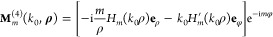

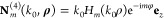
3In eq 3, *H*_*m*_ is the Hankel function of the second kind—the superscript (2) has been omitted for simplicity—and  is the derivative of *H*_*m*_ with respect to its argument. On the right-hand side of eq 2, the term that involves the  CVWF represents the transverse electric (TE) solution, while the term that involves the  CVWF represents the transverse magnetic (TM) solution. For TE scattering, *m* = 0 gives the magnetic dipolar (MD) response, *m* = ±1 the electric dipolar (ED) response, and *m* = ±2 the electric quadrupolar (EQ) response; for TM scattering, the ED, MD, and MQ contributions are given by *m* = 0, *m* = ±1, and *m* = ±2, respectively.^21^ We aim to calculate *A*_*m*_ and *B*_*m*_ to fully determine the multipole response.

Next, we employ a global polar coordinate system, O*xy*, as depicted in Figure 1b. Multiplying **E**^sc^(**ρ**) by exp(i*m*′φ), integrating on the circular circumference *C* of radius ρ that encloses the scatterer *S*, and utilizing the orthogonality relation of the exponential functions, *A*_*m*_ and *B*_*m*_ are expressed via contour integrals of the components of **E**^sc^(**ρ**), i.e.,
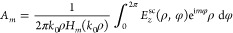

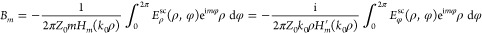
4As is evident, *B*_*m*_ can be evaluated either via  or . To evaluate the components of **E**^sc^(**ρ**), the 2-D scattering problem is formulated by the EFVIE,^41^ namely,
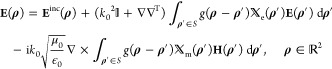
5In eq 5, T denotes transposition, *g*(**ρ** – **ρ**′) = −i/4*H*_0_(*k*_0_|**ρ** – **ρ**′|) is the 2-D free space GF,^45^ and  and  are the normalized tensorial electric and magnetic contrast functions. When **ρ** ∈ *S*_0_, the two 2-D volumetric integrals in eq 5 represent **E**^sc^(**ρ**); introducing the 2-D volumetric current densities **J**_eq_(**ρ**) and **M**_eq_(**ρ**), one writes
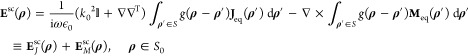
6In what follows, we proceed separately with the evaluation of  and .

To evaluate , the tensorial expansion  of the 2-D GF *g*(**ρ** – **ρ**′) is used, given by eq 6 of ref (41). Since the 2-D volumetric integrals in eq 6 are evaluated for **ρ**′ ∈ *S* and because **ρ** ∈ *S*_0_, the branch ρ > ρ′ is used for . This branch has the tensorial expansion
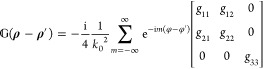
7where *g*_11_ = *H*_*m*_(*u*)*J*_*m*_(*v*) + *k*_0_^2^*H*_*m*_^′^(*u*)*J*_*m*_^′^(*v*), *g*_12_ = *H*_*m*_(*u*)*J*_*m*_^′^(*v*) + *H*_*m*_^′^(*u*)*J*_*m*_(*v*), *g*_21_ = −*H*_*m*_^′^(*u*)*J*_*m*_(*v*) – *H*_*m*_(*u*)*J*_*m*_^′^(*v*), *g*_22_ = *H*_*m*_(*u*)*J*_*m*_(*v*) + *k*_0_^2^*H*_*m*_^′^(*u*)*J*_*m*_^′^(*v*), *g*_33_ = *k*_0_^2^*H*_*m*_(*u*)*J*_*m*_(*v*), *u* = *k*_0_ρ, *J*_*m*_ is the Bessel function,  is the derivative of *J*_*m*_ with respect to its argument, and *v* = *k*_0_ρ′. Substituting eq 7 into the  term of eq 6, expressing the  operator in cylindrical coordinates, and applying it under the integral sign on the unprimed coordinates, the *z* component of  is given by
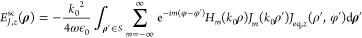
8Substituting eq 8 into the expression for *A*_m_ in eq 4 and utilizing the orthogonality relation of the exponential functions, we get
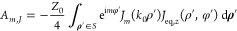
9The φ component of  is given by
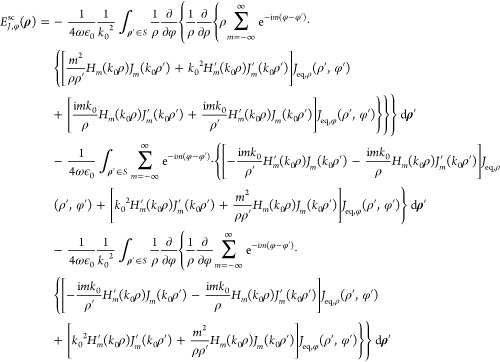
10Substituting eq 10 into the second expression for *B*_*m*_ in eq 4, utilizing the orthogonality relation of the exponential functions and Bessel’s differential equation *u*^2^*H*_*m*_^″^(*u*) + *uH*_*m*_^′^(*u*) + (*u*^2^ – *m*^2^)*H*_*m*_(*u*) = 0—where  is the second derivative of *H*_*m*_ with respect to its argument—after lengthy manipulations and cancellation of terms, we get the elegant result
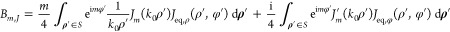
11

To evaluate , we substitute eq 7 into the respective term of eq 6, express the ∇× operator in cylindrical coordinates, and apply it under the integral sign on the unprimed coordinates. This procedure yields
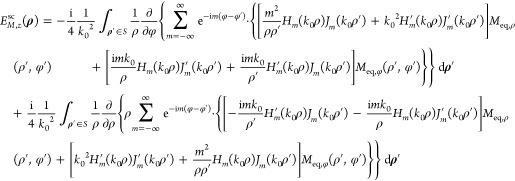
12and
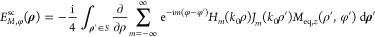
13Employing eq 4, after lengthy manipulations we finally get
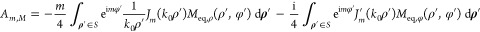
14and
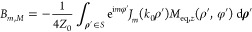
15

The complete solution for the expansion coefficients *A*_*m*_ and *B*_*m*_ is obtained by combining eqs 9, 11, 14, and 15 as
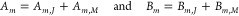
16Inspection of eqs 9, 11, 14, and 15 reveals that *B*_*m*_ can be obtained from *A*_*m*_, and *vice versa*, using the duality schemes *A*_*m*_ → *B*_*m*_, *J*_eq,*z*_ → *M*_eq,*z*_, *M*_eq,ρ_ → −*J*_eq,ρ_, *M*_eq,φ_ → −*J*_eq,φ_ and *B*_*m*_ → −*A*_*m*_, *M*_eq,*z*_ → −*J*_eq,*z*_, *J*_eq,ρ_ → *M*_eq,ρ_, *J*_eq,φ_ → *M*_eq,φ_, while always ϵ_0_ ↔ μ_0_.

### Multipole Decomposition for a Collection of Scatterers

In this case, the configuration comprises *N* scatterers located in free space, as depicted in Figure 1c. Defining a global polar coordinate system O*xy*, each scatterer occupies domain *S*_*j*_ (*j* = 1, 2, ..., *N*) and is characterized by a gyrotropic material having **ϵ**_*j*_(**ρ**), **μ**_*j*_(**ρ**) (*j* = 1, 2, ..., *N*). The background medium *S*_0_ is free space. Upon external excitation, the 2-D volumetric current densities **J**_eq,*j*_(**ρ**) and **M**_eq,*j*_(**ρ**) (**ρ** ∈ *S*_*j*_, *j* = 1, 2, ..., *N*) are induced. The collective response of all equivalent current densities produces the scattered field **E**^sc^(**ρ**) in *S*_0_, i.e.,
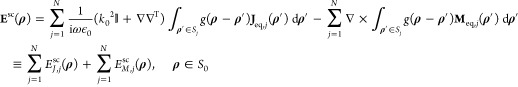
17Therefore, the expansion coefficients *A*_*m*_ and *B*_*m*_ of the system, i.e., with respect to the O*xy* coordinate system, are obtained by
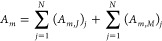

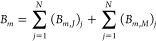
18where , ,  and  are computed using eqs 9, 11, 14, and 15, respectively, for *j* = 1, 2, ..., *N*.

### Scattering Width, Scattering Cross Section, Absorption Cross Section, and Extinction Cross Section

By introducing the dimensionless quantities
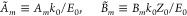
19where *E*_0_ is the amplitude of **E**^sc^(**ρ**), the latter is written in the form
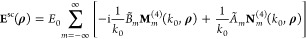
20In the far field (ρ → *∞*), , where **f**(φ) is the scattering amplitude. Then the scattering width σ(φ) (0 ≤ φ < 2π) is given by^43^
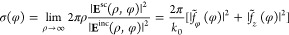
21where  and  are defined via **f**(φ) = 1/[*f̃*_φ_(φ)**e**_φ_ + *f̃*_*z*_(φ)**e**_*z*_] ≡ 1/**f̃**(φ), with
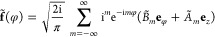
22while we have assumed that |**E**^inc^(ρ, φ)|^2^ = 1. Equations 9, 11, 14, 15, 16, 21, and 22 allow us to calculate the multipole contributions to σ(φ). When the TE and TM separation is valid,  corresponds to the TE scattering and  to the TM scattering.

The full-wave scattering cross section is given by
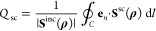
23where **e**_*n*_ is the outward normal unit vector on the contour *C* enclosing all scatterers—see Figure 1b for the case of one scatterer—while **S**^inc^ and **S**^sc^ are the incident and scattered far-field time-averaged power flows, with  and *E*_0_ = 1 V/m. Calculating *Q*_sc_ versus frequency gives the spectrum of the configuration. The multipole decomposition of *Q*_sc_ is obtained via
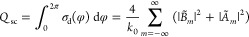
24where  is the differential scattering cross section. The *Q*_sc_ for TE scattering is calculated solely via  and that for TM scattering via . For TE scattering, the *m* = 0 term in eq 24 gives the MD response, the *m* = ±1 terms give the ED response, and the *m* = ±2 terms give the EQ response to the spectrum. For TM scattering, the ED, MD, and MQ responses to the spectrum are calculated by keeping the *m* = 0, *m* = ±1, and *m* = ±2 terms, respectively, in eq 24.

Finally, to discuss the specifics of the scattering from the point of view of energy relations, we introduce the absorption cross section *Q*_abs_, whose full-wave evaluation is given by
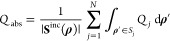
25where *Q*_*j*_ is the power loss density of each domain *S*_*j*_. Therefore, the full-wave extinction cross section is given by *Q*_ext_ = *Q*_sc_ + *Q*_abs_.^46^ Its multipole decomposition for TE scattering is obtained via^46^
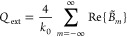
26where Re denotes the real part. For TM scattering  is replaced by  in eq 26.

## Validation

Herein we validate the developed theory by calculating the multipole decompositions of various 2-D structures and compare the results with those of alternative formulations. In particular, the present theory is combined with COMSOL, which we use to compute the full-wave *Q*_sc_ spectrum versus the operating frequency *f* via eq 23 and its multipole contributions using eqs 24, 19, 16, 15, 14, 11, and 9. Then we compare our results with the exact formulations for isotropic and gyrotropic circular cylinders,^39^ with the Mathieu functions method for elliptical cylinders,^40^ with the CFVIE-CDSE for core–shell circular cylinders,^41^ and with the MAS for circular core–elliptical shell and circular dimer cylinders.^42^

**Figure 2:**
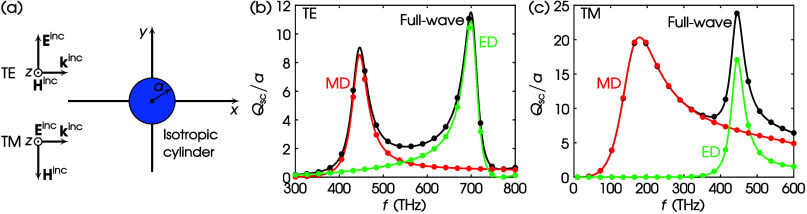
(a) Scattering by an isotropic circular cylinder. Values of parameters: ϵ_1r_ = ϵ_3r_ = 25, ϵ_2r_ = 0, μ_1r_ = μ_3r_ = 1, μ_2r_ = 0, *a* = 50 nm. (b) TE scattering. (c) TM scattering. Curves: exact; dots: this work via COMSOL; black: full-wave; red: MD; green: ED.

At first, we focus on the visible and near-IR parts of the spectrum. In Figure 2a, we assume a high-index dielectric cylinder of circular cross section. The values of the parameters are given in the figure caption. We calculate the normalized full-wave scattering cross section, i.e., *Q*_sc_/*a*, using the exact solution^39^ under TE illumination, as shown in Figure 2b by the black curve. The multipole decomposition is inherent in the analytical formulation, and the red and green curves show the corresponding MD and ED contributions, respectively. The calculated full-wave *Q*_sc_/*a* from eq 23—with the aid of COMSOL—is depicted with black dots, while the corresponding multipole decomposition, using the methodology proposed here, is depicted by red dots for MD and green dots for ED. The agreement between the exact calculation and the multipole decomposition of the developed theory is apparent. The agreement is also true for TM illumination, as shown in Figure 2c.

**Figure 3:**
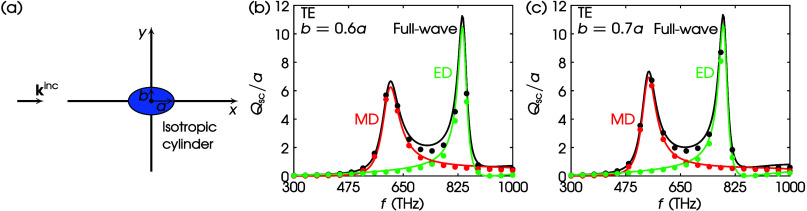
(a) TE scattering by an isotropic elliptical cylinder. Values of parameters: ϵ_1r_ = ϵ_3r_ = 25, ϵ_2r_ = 0, μ_1r_ = μ_3r_ = 1, μ_2r_ = 0, *a* = 50 nm. (b) *b* = 0.6*a*. (c) *b* = 0.7*a*. Curves, Mathieu functions method; dots, this work via COMSOL; black, full-wave; red, MD; green, ED.

Next, we assume a high-index dielectric cylinder with an elliptical cross section, as shown in Figure 3a. The semimajor and semiminor axes are *a* and *b*, respectively. We keep *a* = 20 μm and consider two different values for *b*, i.e., *b* = 0.6*a* and *b* = 0.7*a*. We assumed TE illumination for both cases. In Figure 3b, we depict the *Q*_sc_/*a* spectrum for *b* = 0.6*a*. The black curve corresponds to the full-wave *Q*_sc_/*a* using the Mathieu functions method,^40^ which can be decomposed to its MD—red curve—and ED—green curve—contributions. The corresponding full-wave *Q*_sc_/*a* via eq 23 is shown by black dots, while the result of the proposed multipole decomposition is shown by red dots for MD and green dots for ED. The proposed theory matches perfectly the one from ref (40). In Figure 3c, we show the respective results for the system with *b* = 0.7*a*.

**Figure 4:**
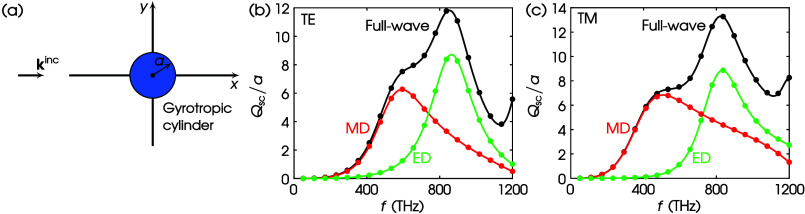
(a) Scattering by a gyrotropic circular cylinder. Values of parameters: ϵ_1r_ = 4, ϵ_2r_ = 1, ϵ_3r_ = 5, μ_1r_ = 2, μ_2r_ = 0.5, μ_3r_ = 3, *a* = 50 nm. (b) TE scattering. (c) TM scattering. Curves, exact; dots, this work via COMSOL; black, full-wave; red, MD; green, ED.

In Figure 4a, we depict a cylinder with a circular cross section consisting of gyrotropic material, i.e., both the permittivity and permeability are tensors. In Figure 4b, we plot the full-wave *Q*_sc_/*a* for TE illumination, calculated using the exact solution—black curve—and the result from eq 23—black dots. We also plot the constituent dipolar contributions, i.e., MD—red curve/dots—and ED—green curve/dots. It is apparent that the agreement between the exact solution and the proposed theory is excellent. In Figure 4c, we repeat the calculation for TM illumination. Still, the agreement remains.

**Figure 5:**
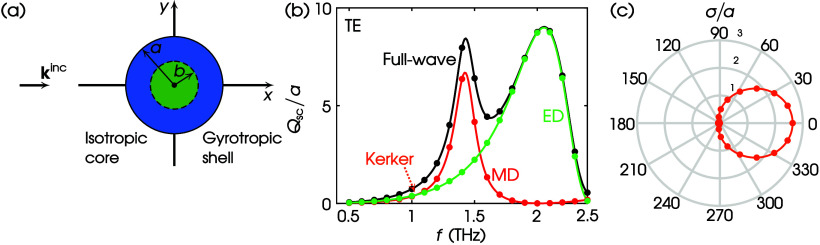
(a) TE scattering by a core–shell circular cylinder. Values of parameters: ϵ_cr_ = 25, μ_cr_ = 1, ϵ_1r_ = 4, ϵ_2r_ = 1, ϵ_3r_ = 5, μ_1r_ = 2, μ_2r_ = 0.5, μ_3r_ = 3, *a* = 20 μm, *b*/*a* = 0.75. (b) *Q*_sc_/*a* spectrum. Curves: CFVIE-CDSE; dots: this work via COMSOL; black: full-wave; red: MD; green: ED. Orange dotted arrow: location of the first MD-ED intersection at *f* = 1.0389 THz. (c) σ/*a* at *f* = 1.0389 THz. Orange curve: CFVIE-CDSE; orange dots: this work via COMSOL.

Next, we focus our study on the THz spectrum of frequencies. We proceed with an inhomogeneous core–shell cylinder of circular cross section as shown in Figure 5a, consisting of a high-index dielectric core with radius *b*, relative permittivity ϵ_cr_, and relative permeability μ_cr_ coated with a gyrotropic shell of outer radius *a*. The full-wave *Q*_sc_/*a* for the TE illumination is calculated using the CFVIE-CDSE.^41^ Results are shown by the black curve in Figure 5b. In contrast, the colored curves depict the corresponding MD—red—and ED—green—contributions. The multipole decomposition is shown by dots using the respective colors. It is evident that all of the results agree perfectly. In addition, in Figure 5c we demonstrate the Kerker effect,^33^ i.e., zero backscattering (first Kerker point^47^), by plotting the full-wave normalized scattering width, i.e., σ/*a*, as computed by the CFVIE-CDSE and COMSOL. The Kerker effect takes place at *f* = 1.0389 THz, as shown by the orange dotted arrow in Figure 5b, where the MD and ED contributions intersect. The two methods in Figure 5c perfectly agree, showing zero backscattering, thus further verifying the validity of our theory.

**Figure 6:**
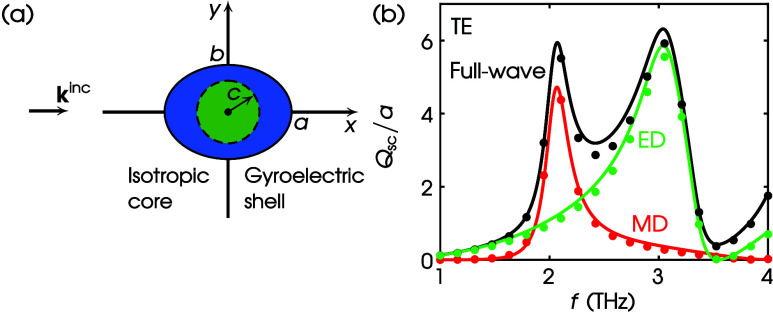
(a) TE scattering by a core–shell circular–elliptical cylinder. Values of parameters: ϵ_cr_ = 25, μ_cr_ = 1, ϵ_1r_ = 4, ϵ_2r_ = 1, ϵ_3r_ = 5, μ_1r_ = 1, μ_2r_ = 0, μ_3r_ = 1, *a* = 20 μm, *c*/*a* = 0.5, *b*/*a* = 0.8. (b) *Q*_sc_/*a* spectrum. Curves, MAS; dots, this work via COMSOL; black, full-wave; red, MD; green, ED.

In Figure 6a we depict a core–shell cylinder consisting of a high-index dielectric *c*-radius circular core of relative permittivity ϵ_cr_ and relative permeability μ_cr_, coated by a gyroelectric shell of elliptical cross section of semimajor axis *a* and semiminor axis *b*. Light impinges along the major axis of the ellipse in the TE polarization. To calculate the full-wave *Q*_sc_/*a*, shown by the black curve in Figure 6b, we employ the MAS.^42^ The corresponding multipole decomposition via the MAS is shown by red—MD—and green—ED—curves. In addition, the black dots correspond to the full-wave *Q*_sc_/*a* as computed by eq 23, while the red and green dots correspond to the multipole decomposition using the proposed theory. The MAS and the proposed theory behave similarly, and the agreement is satisfactory.

**Figure 7:**
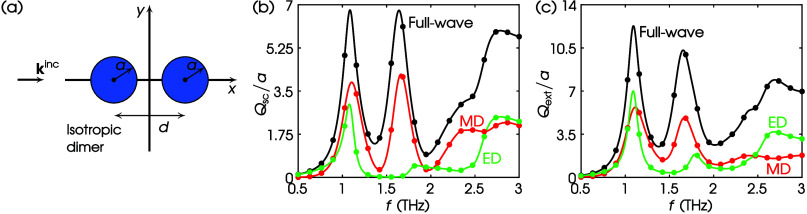
(a) TE scattering by a dimer. Values of parameters: ϵ_r_ = 25 – 2i, μ_r_ = 1, *a* = 20 μm, *d* = 60 μm. (b) *Q*_sc_/*a* spectrum. Curves, MAS; dots, this work via COMSOL; black, full-wave; red, MD; green, ED. (c) Same as (b) but for *Q*_ext_/*a*.

Until now, we have examined structures constituted by sole cylinders, but the theory also holds for a collection of such. As an example, we assume the dimer shown in Figure 7a. Two identical high-index lossy dielectric cylinders of permittivity ϵ_r_ and permeability μ_r_ are placed with a center-to-center distance *d*. Light impinges along the *x* axis with TE polarization, as shown in Figure 7a. The full-wave *Q*_sc_/*a* of the dimer is calculated using MAS, and it is depicted by the black curve in Figure 7b. The multipole decomposition via the MAS is shown by red—MD—and green—ED—curves. The corresponding full-wave *Q*_sc_/*a* via eq 23 is shown by black dots, and the multipole decomposition via the proposed theory by colored symbols that match perfectly the MAS solution. Since the cylinders are lossy, in Figure 7c we further calculate the full-wave and multipole decomposition quantities for the *Q*_ext_/*a*. In particular, the full-wave *Q*_ext_/*a* in COMSOL is computed by *Q*_ext_/*a* = *Q*_sc_/*a* + *Q*_abs_/*a* via eqs 23 and 25, while the *Q*_ext_/*a* multipole decomposition from the proposed theory is computed via eq 26. It is evident that all quantities are in agreement.

The examples presented in this section confirm that the proposed theory can accurately decompose the scattered field into its magnetic and electric dipolar components, providing critical insights into the scattering mechanisms for both TE and TM illuminations. This validation suggests that the method can be reliably applied to other, more complex structures to interpret their EM scattering behavior.

## Application on Oligomer-Based Highly Directional Switching Using Active Media

**Figure 8:**
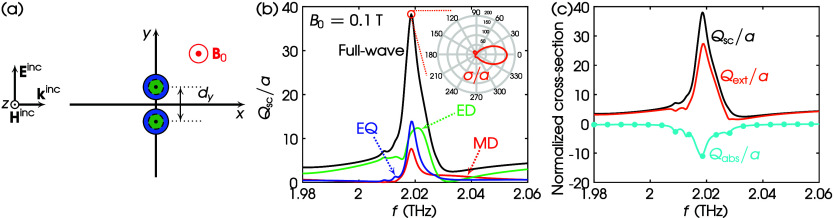
(a) TE scattering by a high-index core–MO shell dimer. Values of parameters: ϵ_cr_ = 25, μ_cr_ = 1, MO shell, *a* = 20 μm, *b*/*a* = 0.75, and *d*_*y*_ = 60 μm. (b) *Q*_sc_/*a* spectrum for *B*_0_ = 0.1 T and α = −0.001 (active). Black, full-wave; red, MD; green, ED; blue, EQ. Inset: σ/*a* at *f* = 2.0186 THz. (c) *Q*_sc_/*a*, *Q*_abs_/*a*, and *Q*_ext_/*a* spectra for the same values of parameters as in (b). Black, full-wave *Q*_sc_/*a* as computed by eq 23; cyan curve, full-wave *Q*_abs_/*a* as computed by eq 25; orange, full-wave *Q*_ext_/*a* as computed by eq 26; cyan dots, *Q*_ext_/*a* – *Q*_sc_/*a*.

The developed framework is employed to study an emerging photonics application on oligomer-based highly directional switching using active media. In Figure 8a, we employ a dimer whose elements are made of core–shell cylinders with a center-to-center distance *d*_*y*_ = 60 μm. Each core has radius *b* = 15 μm and consists of a high-index dielectric material of relative permittivity ϵ_cr_ = 25 and relative permeability μ_cr_ = 1. Each shell has an outer radius *a* = 20 μm and consists of an active magneto-optical (MO) medium which is tunable under an external magnetic flux density **B**_0_ = *B*_0_**e**_*z*_. To this end, we use indium antimonide (InSb) whose gyroelectric permittivity is given by eq 1 with^48^
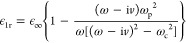

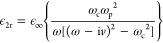

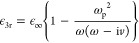
In these definitions, ϵ_*∞*_ = 15.6 accounts for interband transitions, ω_p_ = [*N*_e_*e*^2^/(ϵ_0_ϵ_∞_m*)]^1/2^ = 4π × 10^12^ rad s^–1^ is the plasma angular frequency (where *N*_e_ is the electron density, *e* is the elementary charge, and m* = 0.0142*m*_e_ is electron’s effective mass, where *m*_e_ is the electron’s rest mass), ω_c_ = *eB*_0_/m* is the cyclotron angular frequency, and *v* = αω_p_ is the damping angular frequency, where α is a dimensionless parameter. Based on the adopted time dependence exp(iω*t*), the MO material is passive (lossy) for α > 0 and active (exhibits gain) for α < 0. Therefore, by assuming the parameters of InSb, a passive material, we set negative values to the parameter α and made it active. In Figure 8b, we plot the full-wave *Q*_sc_/*a* spectrum and the multipole decomposition when an external *B*_0_ = 0.1 T is applied and active shells are used in the dimer with α = −0.001. Due to the external bias, a directional-scattering mode^11^ is induced at *f* = 2.0186 THz, whose peak is shown by the orange circle. The multipole decomposition reveals that this mode is due to a collective contribution between the MD and EQ responses that resonate at the same frequency. The phenomenon is further enhanced by the ED response, which is almost resonant at the same frequency. Precisely at the peak, we plot the full-wave normalized scattering width, i.e., σ/*a*, as computed by COMSOL. This full-wave simulation is depicted in the inset of Figure 8b, where the directional forward scattering is shown. Since the structure in Figure 8a incorporates an active medium, we discuss the specifics of the scattering from the point of view of energy relations by calculating the absorption and extinction cross sections. In Figure 8c, we plot the full-wave *Q*_sc_/*a*, the full-wave *Q*_abs_/*a*, and the full-wave *Q*_ext_/*a* spectra, as computed by eqs 23, 25, and 26, respectively. The difference *Q*_ext_/*a* – *Q*_sc_/*a*, shown by the cyan dots in Figure 8c, is in perfect agreement with the *Q*_abs_/*a*, shown by the cyan curve in Figure 8c, proving the correctness of the calculations. *Q*_abs_/*a* is negative, indicating emission due to the active layers. Therefore, the energy scattered by the cylinder equals the energy provided by the source via the incident plane wave plus the energy due to the emission. In the spectral window of Figure 8c, the emerging mode at *f* = 2.0186 THz exhibits the maximum gain.

**Figure 9:**
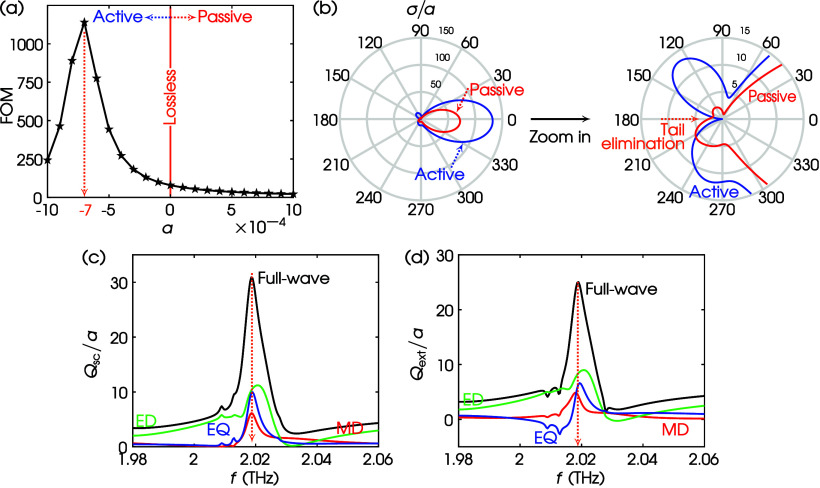
(a) FOM vs α for the dimer of Figure 8a for *B*_0_ = 0.1 T and *f* = 2.0186 THz. (b) σ/*a* at α = ∓7 × 10^–4^, *B*_0_ = 0.1 T, and *f* = 2.0186 THz. Blue, α = −7 × 10^–4^ (active); red, α = +7 × 10^–4^ (passive). Zoom-in: tail elimination at backscattering. (c) *Q*_sc_/*a* spectrum for the same values of parameters as in (b) using α = −7 × 10^–4^. Black, full-wave; red, MD; green:, ED; blue, EQ. Orange dotted arrow: location of *f* = 2.0186 THz. (d) Same as (c) but for the *Q*_ext_/*a* spectrum.

To reveal the advantage of using active shells in the dimer rather than passive ones, we define the figure of merit (FOM) as the ratio of the forward scattering to the backscattering, i.e.,
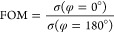
27In Figure 9a, we plot the FOM versus various values of the dimensionless parameter α. There exists a specific value α = −0.0007 where the FOM is maximized. In Figure 9b, we plot σ/*a* using this specific value of α as well as its opposite value, i.e., α = +0.0007. As revealed by the zoom-in, the active shell medium (α = −0.0007) eliminates the tail in the backscattering direction, while the passive shell medium (α = +0.0007) does not. Such tail suppression to obtain directionality is also expected in lossy structures that are illuminated using complex-frequency waves, exhibiting the so-called *virtual gain*, as shown in recent publications.^49−51^

To further explain the origin of the FOM maximization that yields a backscattering suppression at *f* = 2.0186 THz when α = −0.0007, we plot in Figure 9c,d the full-wave *Q*_sc_/*a* and *Q*_ext_/*a* spectra and their multipole decompositions. As is evident, since the structure of the dimer operates above the subwavelength regime, higher-order multipoles contribute in the construction of the emerging forward-scattering mode. Specifically at *f* = 2.0186 THz, the FOM maximization—featuring the backscattering suppression—is primarily contributed by the interference of the ED and EQ terms, with a secondary confluence from the MD term. These multipole terms interfere destructively toward the backscattering direction of Figure 9b, producing the FOM maximization.

**Figure 10:**
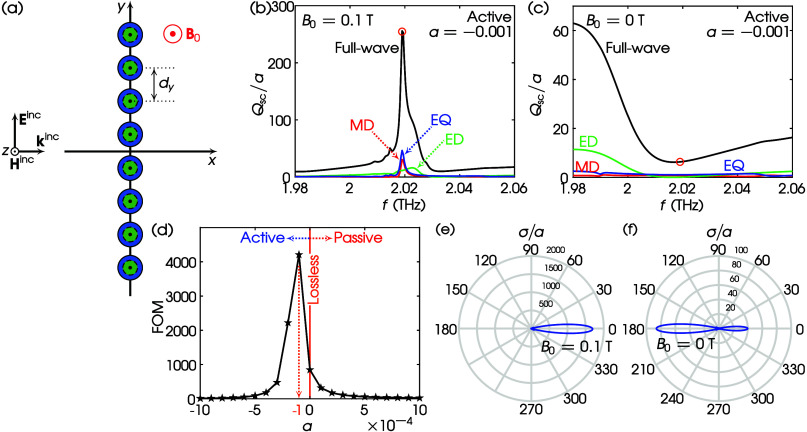
(a) TE scattering by a high-index core–MO shell octamer. Values of parameters: ϵ_cr_ = 25, μ_cr_ = 1, MO shell, *a* = 20 μm, *b*/*a* = 0.75, and *d*_*y*_ = 60 μm. (b) *Q*_sc_/*a* spectrum for *B*_0_ = 0.1 T and α = −0.001 (active). Black, full-wave; red, MD; green, ED; blue, EQ. Orange circle: resonant mode at *f* = 2.0190 THz. (c) Same as (b) but for *B*_0_ = 0 T. Orange circle: scattering at *f* = 2.0190 THz. (d) FOM vs α for the octamer of (a) at *B*_0_ = 0.1 T and *f* = 2.0190 THz. (e) σ/*a* at α = −1 × 10^–4^ (active), *B*_0_ = 0.1 T, and *f* = 2.0190 THz. (f) Same as (e) but for *B*_0_ = 0 T.

To enhance the scattering and make it highly directive, we examine in Figure 10a a core–shell octamer having the same unit structure and values of parameters as the dimer of Figure 8a. Using active shells (α = −0.001), in Figure 10b,c we plot the *Q*_sc_/*a* spectra and the multipole decompositions for *B*_0_ = 0.1 and 0 T. Due to the electrically large size of the octamer, higher-order multipole responses contribute to the spectra; however, these are not depicted for graph clarity. Now, the peak (depicted by the orange circle in Figure 10b) takes place at *f* = 2.0190 THz, which is still a collective contribution of at least the MD and EQ responses. For *B*_0_ = 0 T, smaller scattering arises at the same frequency (depicted by the orange circle in Figure 10c).

Finally, to optimize the forward scattering of the octamer’s resonant mode, in Figure 10d we show that for α = −0.0001, the FOM becomes maximum. With this value of α, in Figure 10e we plot σ/*a* when the magnetic bias is in the on state. A highly directional lobe is revealed at the forward scattering with tail elimination at the backscattering. When the magnetic bias is in the off state, Figure 10f shows backscattering with remaining forward scattering. Nevertheless, this on–off state suggests that a core–shell oligomer with active medium shells can operate as a highly directional active switching device in contemporary photonics.

## Conclusion

In this work, we developed and validated a comprehensive EM multipole decomposition framework for 2-D structures with a specific focus on inhomogeneous and anisotropic cylindrical scatterers. Our method leverages the expansion of scattered fields using divergenceless CVWFs and employs 2-D volumetric integrals to express the unknown expansion coefficients. Our results on multipole decomposition using finite element simulations were validated by comparing our results with analytical and numerical methods that provide inherently the multipole decomposition. Furthermore, we demonstrated the applicability of the developed framework by analyzing photonic applications on oligomer-based highly directional scattering switching using active MO media. Our findings reveal that the collective contributions of magnetic and electric multipole responses can be harnessed to achieve highly directional forward scattering, a promising avenue for future photonic devices.
